# Cytogenetic and molecular abnormalities in chronic myelomonocytic leukemia

**DOI:** 10.1038/bcj.2016.5

**Published:** 2016-02-05

**Authors:** M M Patnaik, A Tefferi

**Affiliations:** 1 Division of Hematology, Mayo Clinic, Rochester, MN, USA

## Abstract

Chronic myelomonocytic leukemia (CMML) is a clonal stem cell disorder associated with peripheral blood monocytosis and an inherent tendency to transform to acute myeloid leukemia. CMML has overlapping features of myelodysplastic syndromes and myeloproliferative neoplasms. Clonal cytogenetic changes are seen in ~30%, whereas gene mutations are seen in >90% of patients. Common cytogenetic abnormalities include; trisomy 8, -Y, -7/del(7q), trisomy 21 and del(20q), with the Mayo–French risk stratification effectively risk stratifying patients based on cytogenetic abnormalities. Gene mutations frequently involve epigenetic regulators (*TET2* ~60%), modulators of chromatin (*ASXL1* ~40%), spliceosome components (*SRSF2* ~50%), transcription factors (*RUNX1* ~15%) and signal pathways (*RAS* ~30%, *CBL* ~15%). Of these, thus far, only nonsense and frameshift *ASXL1* mutations have been shown to negatively impact overall survival. This has resulted in the development of contemporary, molecularly integrated (inclusive of *ASXL1* mutations) CMML prognostic models, including Molecular Mayo Model and the Groupe Français des Myélodysplasies model. Better understanding of the prevalent genetic and epigenetic dysregulation has resulted in emerging targeted treatment options for some patients. The development of an integrated (cytogenetic and molecular) prognostic model along with CMML-specific response assessment criteria are much needed future goals.

## Introduction

Chronic myelomonocytic leukemia (CMML) is a clonal stem cell disorder with overlapping features of myelodysplastic syndromes (MDS) and myeloproliferative neoplasms (MPN).^1, 2^ CMML often results in peripheral blood monocytosis and has an inherent tendency to transform to acute myeloid leukemia (AML; ~30%).^2, 3^ Clonal cytogenetic changes are seen in ~30% of patients,^4, 5^ whereas molecular and epigenetic abnormalities are seen in >90%.^6, 7^ CMML is further subclassified into CMML-1 (<5% circulating blasts and <10% bone marrow (BM) blasts) and CMML-2 (5–19% circulating blasts, 10–19% BM blasts or when Auer rods are present irrespective of the blast count),^6, 8, 9^ with approximate median overall survival (OS) of 38 and 24 months, respectively.^6, 7^

Gene mutations in CMML involve epigenetic regulators (*TET2* ~60%), chromatin/histone modulators (*ASXL1* ~40%), spliceosome components (*SRSF2* ~50%), transcription factors (*RUNX1* ~15%) and cell signaling (*RAS* ~30%, *CBL* ~15%).^2, 6, 7, 10^ Among these, thus far, on multivariable analyses that have included additional CMML relevant factors, only *ASXL1* mutations (frameshift and nonsense) have been shown to be prognostically detrimental.^6, 7^ This has led to the incorporation of *ASXL1* mutations into molecular prognostic models, such as the Molecular Mayo Model and the Groupe Francais des Myelodysplasies (GFM) model.^6, 7^ In the current review, we discuss and summarize the prevalence, phenotypic, prognostic and therapeutic impact of cytogenetic and molecular abnormalities in CMML.

## Cytogenetic abnormalities in CMML

The 2008 World Health Organization (WHO) criteria define CMML as a disorder characterized by: (a) persistent peripheral blood monocytosis >1 × 10^9^/l, (b) absence of the Philadelphia chromosome and the *BCR-ABL1* fusion oncogene, (c) absence of the *PDGFRA* or *PDGFRB* gene rearrangements, (d) <20% blasts and promonocytes in the peripheral blood and BM, and (e) dysplasia involving one or more myeloid lineages.^1^ If myelodysplasia is absent or minimal, the diagnosis of CMML can still be made if the other requirements are met and an acquired, clonal or molecular genetic abnormality is present in the hematopoietic cells or if the monocytosis has persisted for at least 3 months and other causes of monocytosis have been excluded.^1, 2^

The *BCR-ABL1* fusion oncogene defines chronic myeloid leukemia, a unique myeloid neoplasm in which monocytosis is uncommon.^11^ The platelet-derived growth factor receptors alpha and beta (*PDGFRA*—chromosome *4q12* and *PDGFRB*—chromosome *5q31-q32*) are type III receptor tyrosine kinases. Chromosomal translocations involving *PDGFRA/B* have been associated with myeloid neoplasms characterized by prominent eosinophilia and responsiveness to imatinib.^12, 13^ At times, *PDGFR*-rearranged myeloid neoplasms can be associated with monocytosis and BM dysplasia, but given their unique responsiveness to imatinib, these are no longer classified as CMML. Patients presenting with a clinical phenotype of CMML with eosinophilia should be assessed for the t(5;12)(q31-q32;p13), giving rise to the *ETV6(TEL)-PDGFRB* fusion oncogene.^14^ The association between monocytosis and *PDGFRA* rearrangements is uncommon.^15^

Clonal cytogenetic abnormalities are seen in ~30% of CMML patients.^5, 8, 16, 17^ Common alterations include: trisomy 8 (+8), -Y, abnormalities of chromosome 7 (monosomy 7 and del7q), trisomy 21 (+21), and complex karyotypes (Table 1).^5^ Unlike in MDS, sole del(5q) (<1%) and monosomal karyotypes (~10%) are infrequent.^4, 18, 19^ Based on these findings, the Spanish cytogenetic risk stratification system was developed, categorizing patients into three groups; high risk (trisomy 8, chromosome 7 abnormalities or complex karyotype), intermediate risk (all chromosomal abnormalities, except for those in the high- and low-risk categories), and low risk (normal karyotype or -Y), with 5-year OS of 4, 26 and 35%, respectively.^5^

**Figure 1:**
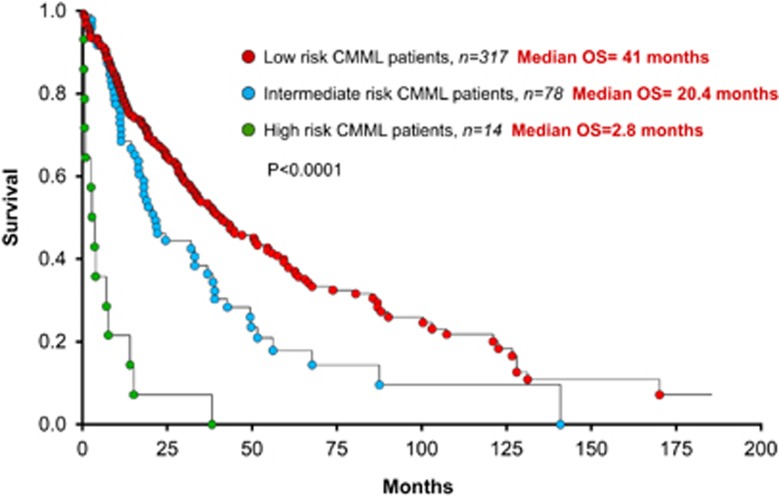
OS of 409 patients with WHO-defined CMML, stratified by the Mayo–French cytogenetic risk stratification system.

Recently, in a large international collaborative study, 409 patients with CMML were analyzed for cytogenetic and molecular abnormalities (268 (66%) and 141 (34%) from the Mayo Clinic and French Consortium, respectively).^4^ Thirty percent displayed an abnormal karyotype, with common abnormalities being +8 (23%), -Y (20%), −7/7q-(14%), 20q- (8%), +21 (8%) and der(3q) (8%).^4^ A step-wise survival analysis resulted in three distinct cytogenetic risk categories: high (complex and monosomal karyotypes), intermediate (all abnormalities not in the high- or low-risk groups) and low (normal, sole -Y and sole der(3q)), with median survivals of 3 (hazard ratio (HR) 8.1, 95% confidence interval (CI) 4.6–14.2), 21 (HR 1.7, 95% CI 1.2–2.3) and 41 months, respectively (Figure 1).^4^ In multivariable analysis, this particular cytogenetic risk stratification remained significant in the context of the Molecular Mayo Model (*P*< 0.0001), MD Anderson prognostic model (*P*<0.0001) and the GFM model (*P*<0.0001) and was effective in predicting leukemic transformation (*P*=0.004).^4^

## Molecular and epigenetic abnormalities in CMML

**Figure 2:**
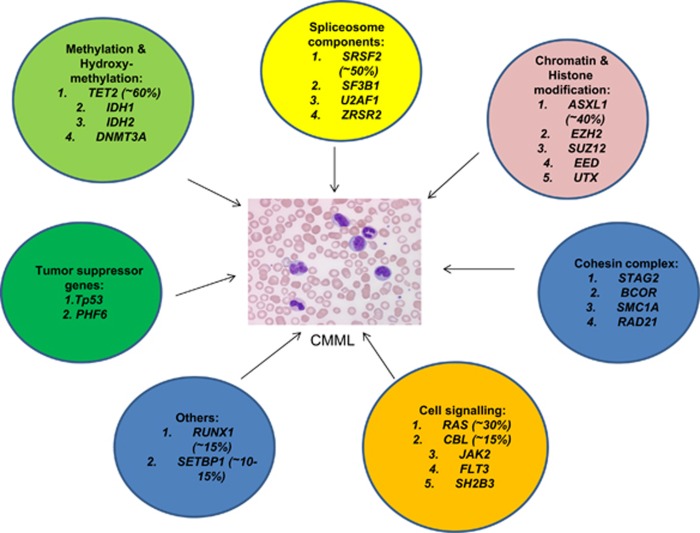
Spectrum of gene mutations seen in patients with CMML.

Gene mutations are seen in >90% of patients with CMML (Figure 2).^20, 21, 22^ These abnormalities can be broadly classified into the following categories:

1. Mutations involving epigenetic regulator genes: *TET2* (~60%), *DNMT3A*, *IDH1*, and *IDH2.*
2. Mutations involving chromatin regulation and histone modification: *ASXL1* (~40%), *EZH2*, *UTX*, *EED*, and *SUZ12.*
3. Mutations involving the splicing machinery (pre-mRNA splicing): *SF3B1*, *SRSF2* (~50%), *U2AF1*, and *ZRSR2.*
4. Mutations involving the cohesin complex: *STAG2*, *BCOR*, *SMC3*, *SMC1A,* and *RAD21.*
5. Mutations involving DNA damage response genes: *Tp53* (~1%) and *PHF6.*
6. Mutations in signal transduction and cellular/receptor tyrosine kinase pathways: *JAK2*, *SH2B3 (LNK)*, *KRAS*, *NRAS* (*RAS* ~30%*)*, *CBL* (~10–15%), *FLT3*, and *NPM1*
7. Others: *RUNX1* (transcription factor) and *SETBP1* (~15%).

**Figure 3:**
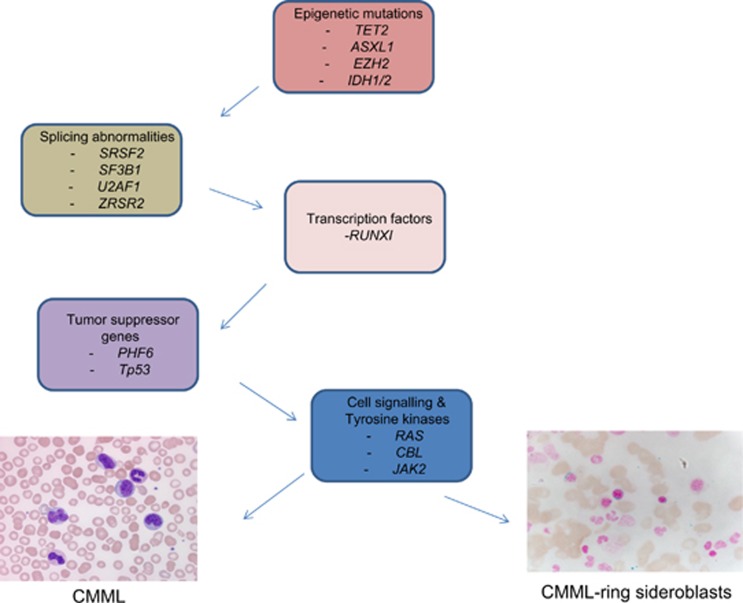
Mutational evolution and clonal hierarchy in patients with CMML.

The genetic heterogeneity of CMML, in patients and in between patients, suggests that the disease has different potential evolutional trajectories.^21, 23^ Current studies suggest that the preferred order of mutational accumulation is *TET2* (or *IDH1/2*) or *ASXL1* (*EZH2*) first, spliceosome component mutations (*SRSF2*, *SF3B1*, *U2AF1 or ZRSR2*) next, followed by transcription factor mutations (*RUNX1*) and then signal pathway gene mutations (*RAS*, *CBL*), inducing GM-CSF (granulocyte macrophage-colony stimulating factor) hypersensitivity and myeloproliferation (Figure 3).^21, 23, 24^

## Mutations in epigenetic regulator genes impacting DNA methylation and hydroxy-methylation (*TET2, DNMT3A, IDH1* and *IDH2*)

*TET2* (*ten-eleven* trans*location* (TET) oncogene family member 2—chromosome 4q24) is a member of the TET family of proteins (TET1–TET3).^25^ *TET2* mutations are seen in ~15% of myeloid neoplasms,^26^ with individual mutational frequencies of; ~60%—CMML, ~15%—MDS, ~15%—polycythemia vera and primary myelofibrosis (PMF), ~20%—secondary AML and ~30%—systemic mastocytosis, with limited prognostic significance.^7, 26, 27, 28^ TET2 has a dioxygenase enzymatic activity and catalyzes the conversion of 5-methyl-cytosine (5-mc) to 5-hydroxy-methyl-cytosine (5-hmc). 5-hmC represents a new base in genomic DNA, which may have a specific effect on transcription and/or may represent an intermediate process in DNA demethylation.^29, 30^ 5-hmC is most often found at transcription start sites and within gene bodies (preferentially in gene exons).^31^

Ko *et al.*^32^ reported that loss of 5-mC was a remarkable characteristic in CMML patients with *TET2* mutations and found 2510 differentially hypomethylated regions and only two hypermethylated regions. In contrast, Figueroa *et al.*^33^ studied *TET2* mutant leukemic cells and identified a hypermethylation phenotype, including 129 differentially methylated regions. Yamazaki *et al.*^25^ using bisulfite pyrosequencing, confirmed that *TET2* mutations affect global methylation in CMML but hypothesized that most of the changes were likely to be outside gene-promoter regions.

In four mouse models, the deletion of *TET2* has resulted in progressive expansion of the hematopoietic progenitor compartment, increased hematopoietic stem cell self-renewal and the progressive development of a proliferative myeloid malignancy, similar to CMML.^34, 35, 36^

Although *TET2* mutations are widely prevalent in CMML (~60%), thus far, they have not been shown to independently impact either OS or leukemia-free survival (LFS).^7^ In a recent study, *TET2* mutations were seen in 46% of CMML patients and the absence of *TET2* mutations negatively impacted OS. Additionally, the presence of clonal *TET2* mutations, in the absence of clonal *ASXL1* mutations (*ASXL1wt/TET2mut*), had a favorable impact on OS.^37^ The mechanism behind this association is unclear. In MDS and younger patients with CMML (age <65 years), the presence of clonal *TET2* mutations, in the absence of clonal *ASXL1* mutations, has been associated with a favorable response to hypomethylating agents (5–azacitidine and decitabine).^38, 39^ Treatment data on this study cohort was incomplete.^37^

**Figure 4:**
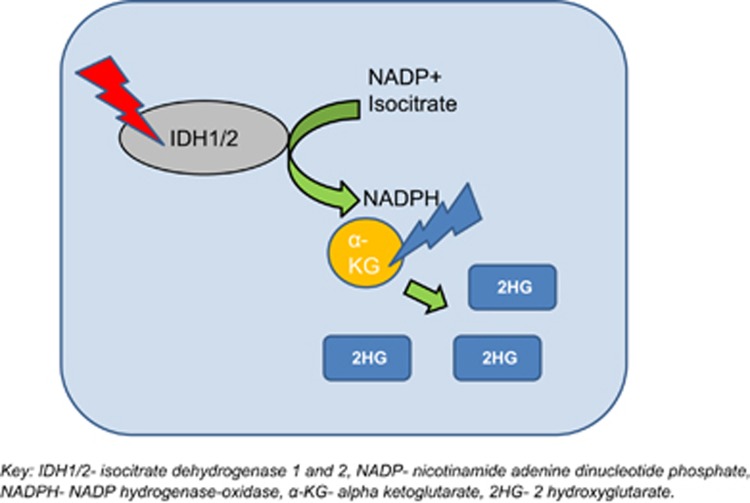
Isocitrate dehydrogenase mutations in CMML.

Mutations involving *IDH1* (isocitrate dehydrogenase—chromosome 2q34) and *IDH2* (chromosome 15q26.1) are uncommon in CMML (<5%) and are mutually exclusive with *TET2* mutations.^7, 40^ IDH1/2 normally participates in the citric acid cycle and converts isocitrate to 5-alpha-ketoglutarate (Figure 4). *IDH* mutations confer a new enzymatic function to these enzymes, resulting in the development of an onco metabolite termed 2-hydroxyglutarate (2-HG).^29^ 2-HG in turn impairs other enzymes, including TET2 and JMJC (Jumonji-C domain containing) family of histone lysine demethylases,^41^ contributing to leukemogenesis.

*DNMT3A* (DNA methyltransferase 3A—chromosome 2p23.3) mutations are seen in MDS (~10%),^42^ cytogenetically normal AML (~30%),^29^ PMF (~10%)^43^ and CMML (<5%).^7^ In CMML, thus far, they have not been shown to impact either OS or LFS.^7^

## Mutations in epigenetic regulator genes impacting chromatin and histone modification (*ASXL1, EZH2, EED, SUZ12* and *UTX*)

**Figure 5:**
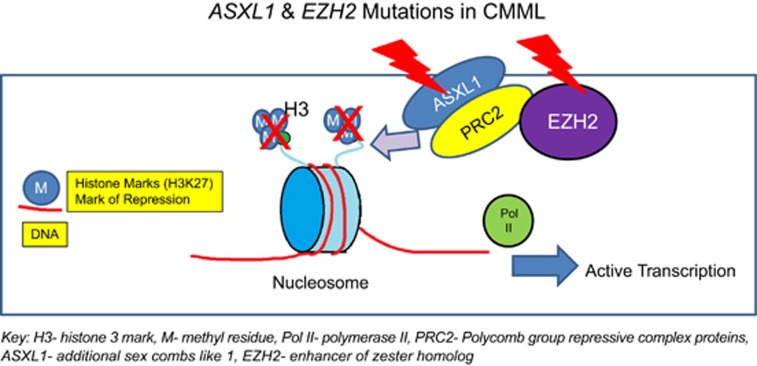
*ASXL1* and *EZH2* mutations in CMML.

The *ASXL1* (additional sex combs-like 1—chromosome 20q11) gene regulates chromatin by interacting with the polycomb group repressive complex proteins (PRC1 and PRC2).^44^ The PRC2 contains histone 3 lysine 27 (H3K27) methyltransferase, EZH2 (enhancer of zeste homolog 2) and its partners EED (embryonic ectoderm development) and SUZ12 (suppressor of zeste 12 homolog) and produces the H3K27 trimethyl mark (Figure 5).^45^ Histone 2A lysine 119 (H2AK119Ub) and H3K27me3 have synergistic roles in PRC-mediated gene repression.^45, 46^ In a seminal paper, Abdel-Wahab *et al.*^46^ demonstrated that *ASXL1* mutations resulted in loss of PRC2-mediated H3K27 trimethylation. In addition, Balasubramani *et al.*^45^ demonstrated that *ASXL1* truncations conferred enhanced activity on the ASXL1-BAP1 (BRCA-associated protein 1) complex. This resulted in global erasure of H2AK119Ub and depletion of H3K27me3, promoting dysregulated transcription.

*ASXL1* mutations are common in myeloid neoplasms, including MDS,^44, 47^ CMML,^7, 9, 48^ PMF^44, 49^ and AML,^47, 50^ with respective mutational frequencies ranging from 15 to 20, 40–50, 20–35 and 5–10%.^20^ In general, they are associated with an aggressive phenotype.^48, 49, 50^ In MDS, Bejar *et al.*^51^ identified *ASXL1* mutations in 63 (14.4%) of 439 MDS patients and found these to be IPSS (International Prognostic Scoring System) independent predictors for shortened OS. In a large (879 patients) PMF collaborative study, *ASXL1* mutations were identified in 20% of patients and were associated with older age, presence of constitutional symptoms, leukocytosis and circulating blasts.^52^ In systemic mastocytosis, *ASXL1* mutations were seen in 9 (14%) of 62 patients and predicted for a shortened OS.^53^ In AML, *ASXL1* mutations have been found to be mutually exclusive with the favorable *NPM1* mutations, with some,^54, 55^ but not all,^56^ studies demonstrating an independent prognostic impact.

In CMML, ~40% of patients carry *ASXL1* mutations, with the most frequent being the c.1934dupG; p.G646WfsX12 (~50%).^7, 9^ Although initially some investigators had considered c.1934dupG; p.G646WfsX12 to be a PCR artefact,^57^ subsequent studies have demonstrated its absence in germ-line DNA and control DNA, establishing it to be a *bona fide* mutation.^20, 58^ In CMML, *ASXL1* mutations are associated with a proliferative phenotype, including higher WBC (white blood counts), higher absolute monocyte count (AMC) and the presence of circulating immature myeloid cells (IMC).^7, 9, 20^

The discovery of gene mutations in CMML has led to their incorporation into prognostic models. A Mayo Clinic study (*n*=226) analyzed several parameters, including *ASXL1* mutations; on multivariable analysis, risk factors for survival included HB (hemoglobulin) <10 gm/dl, platelet count <100 × 10^9^/l, AMC>10 × 10^9^/l and circulating IMC.^9^ *ASXL1* mutations did not impact either the OS or the LFS. The study resulted in the development of the Mayo prognostic model, with three risk categories, low (0 risk factor), intermediate (1 risk factor) and high (⩾2 risk factors), with median survivals of 32, 18.5 and 10 months, respectively.^9^ The GFM demonstrated an adverse prognostic effect for *ASXL1* mutations in 312 patients with CMML; additional risk factors on multivariable analysis included age >65 years, WBC>15 × 10^9^/l, platelet count <100 × 10^9^/l and HB<10 gm/dl in females and <11 gm/dl in males.^7^ The GFM model assigns three adverse points for WBC>15 × 10^9^/l and two adverse points for each one of the remaining risk factors, resulting in a three-tiered risk stratification: low (0–4 points), intermediate (5–7) and high (8–12), with respective median survivals of 56, 27.4 and 9.2 months.^7^ It should be noted that all nucleotide variations (missense, nonsense and frameshift) were regarded as *ASXL1* mutations in the Mayo study,^9^ whereas only nonsense and frameshift *ASXL1* mutations were considered in the French study.^7^

To further clarify the prognostic relevance of *ASXL1* mutations, an international collaborative cohort of 466 CMML patients was analyzed.^4^ In univariate analysis, survival was adversely affected by *ASXL1* (nonsense and frameshift) mutations. In multivariable analysis, *ASXL1* mutations, AMC >10 × 10^9^/l, HB<10 gm/dl, platelets <100 × 10^9^/l and circulating IMC were independently predictive of shortened OS. A regression coefficient-based prognostic model (Molecular Mayo Model) based on these five risk factors delineated high (⩾3 risk factors; HR 6.2, 95% CI 3.7–10.4) intermediate-2 (2 risk factors; HR 3.4, 95% CI 2.0–5.6) intermediate-1 (1 risk factor; HR 1.9, 95% CI 1.1–3.3) and low (no risk factors) risk categories, with median survivals of 16, 31, 59 and 97 months, respectively.^6^

Efficient H3K27 methylation requires the corporation of core components, including EZH2 (catalytic enzyme) and cofactors SUZ12 and EED. The *EZH2* (enhancer of zeste homolog 2—chromosome 7q35-q36) gene, encodes for the PRC2 protein, a highly conserved enzyme that serves as a histone H3K27 methyltransferase (Figure 5). In CMML, *EZH2* mutations are infrequent (~5%) and do not have an independent prognostic impact.^7, 59^ The *UTX* gene (ubiquitously transcribed X chromosome tetratricopeptide repeat—chromosome Xp11.2), encodes a lysine-specific demethylase (6A). *UTX* mutations are seen in ~8% of CMML patients and do not impact survival.^59^

## Spliceosome component mutations (*SRSF2*, *SF3B1*, *U2AF1* and *ZRSR2*)

Spliceosome component mutations (*SRSF2*, *SF3B*, *U2AF1* and *ZRSR2*) affect pre-mRNA splicing.^16, 60^ They are involved in the 3′ splice site recognition of pre-mRNA, including abnormal/alternative splicing. The U2 auxiliary factor that consists of the U2AF65–U2AF1 heterodimer establishes physical interaction with SF1 and a serine/arginine-rich protein such as SRSF1 or SRSF2, resulting in recognition of the 3′ splice site and its nearby polypyrimidine tract.^60^ This leads to the subsequent recruitment of U2 snRNP containing SF3A1 and SF3B1 to establish the splicing A complex.^60^

*SRSF2* (serine/arginine-rich splicing factor 2—chromosome 17q25.2) mutations are seen in patients with MDS, CMML, PMF and AML.^60, 61, 62, 63^ In MDS and PMF, these mutations are seen in ~15–20% of patients and are associated with a shortened OS and LFS.^61, 63, 64^ In CMML, the frequency of *SRSF2* mutations is higher (~50%), and these mutations are associated with increased age, less pronounced anemia and a diploid karyotype.^16^ Mutational hot spots include P95L, P95H and P95R.^16^ Thus far, in CMML, *SRSF2* mutations have not demonstrated an independent prognostic impact on either OS or LFS.^7, 16, 65^

*SF3B1* (splicing factor 3B, subunit 1—chromosome 2q33.1) mutations have a high prevalence (~80%) in patients with MDS and ring sideroblasts (RS)^66^ and can also be seen in patients with CMML and RS (<10%).^16^ In MDS and CMML, these mutations do not influence either the OS or LFS.^63, 67^ The mutational hot spots for *SF3B1* include K700E (~50%), H662Q and K666N.^16, 66^ Gene expression studies have shown that *SF3B1* mutations result in the downregulation of ABCB7 (ATP-binding cassette, sub-family B, member 7), a mitochondrial cassette protein, resulting in the development of RS.^68^

*U2AF1* (U2 small nuclear RNA auxiliary factor—chromosome 21q22) mutations are seen in ~10% of patients with CMML and have thus far lacked an independent prognostic effect.^60^ The mutational hot spots for *U2AF1* include S34F, Q157 and R158H.^16^ *ZRSR2* mutations (zinc finger, RNA-binding motif and serine/arginine-rich factor—chromosome Xp22.2) are very infrequent and once again do not have an independent prognostic impact.^60^

## Mutations involving the cohesin complex (*STAG2, BCOR, SMC3, SMC1A* and *RAD21*)

Cohesin is a multimeric protein complex composed of four core subunits: SMC1, SMC3, RAD21, and STAG proteins, together with a number of regulatory molecules, such as PDS5, NIPBL and ESCO.^69^ Cohesin is thought to be engaged in the cohesion of sister chromatids during cell division, postreplicative DNA repair and the regulation of global gene expression.^70, 71^ Germline mutations in cohesin components lead to the congenital multisystem malformation syndromes known as Cornelia de Lange syndrome and Roberts syndrome.^70, 71^

Mutations involving the cohesin complex can be seen in myeloid neoplasms, with individual mutational frequencies of ~12% AML, ~8% MDS, ~6% chronic myeloid leukemia, ~1% MPN and ~10% in CMML.^69^ These mutations frequently coexist with other myeloid relevant mutations, including *TET2*, *ASXL1* and *EZH2*.^69^ The prognostic impact of these mutations remains to be determined.

## Mutations involving DNA damage response genes (*Tp53* and *PHF6*)

The *PHF6* gene (plant homeodomain finger protein 6—chromosome Xp26.3) is a tumor-suppressor gene commonly mutated in T-cell acute lymphoblastic leukemia (~20%).^72^ *PHF6* has two PHD domains involved in the recognition of epigenetic histone marks, which suggests a role in the epigenetic regulation of gene expression. *PHF6* mutations are infrequent in chronic myeloid neoplasms, including CMML (~2.5%).

The *Tp53* (tumor protein 53—chromosome 17p13.1) gene encodes a tumor-suppressor protein containing transcriptional activation, DNA binding and oligomerization domains. The encoded protein regulates the expression of target genes under stress, thereby inducing cell cycle arrest, apoptosis, senescence, DNA repair and changes in metabolism. Mutations in this gene are associated with a variety of human cancers, including hereditary cancer syndromes such as the Li-Fraumeni syndrome. *Tp53* mutations are very infrequent in CMML (~1%).^7^

## Mutations in transcription factors, signal transduction and cellular/receptor tyrosine kinase pathways (*RUNX1, JAK2, KRAS, NRAS, CBL, SH2B3, FLT3*)

The *RUNX1* gene (runt-related transcription factor 1—chromosome 21q22.3) encodes the DNA-binding, alpha subunit of the core-binding factor and is essential for normal hematopoiesis and differentiation. It helps regulate the expression of G-CSF, interleukin-3, T-cell receptor and myeloperoxidase.^73^ Mutations and translocations involving *RUNX1* have been well characterized in core-binding factor AML (t(8;21)(q22;q22) *RUNX1/RUNX1T1*) and MDS.^73^ In CMML, *RUNX1* mutations are seen in ~15% of patients.^7, 74, 75^ These mutations do not impact OS but can be associated with a shorter LFS, especially in patients with C-terminal mutations.^7, 74, 75^

Signal pathway mutations are common in CMML and include: *JAK2*V617F (~10–15%), *RAS* (*KRAS* and *NRAS* ~20–30%), and *CBL* (~10–20%) mutations.^7, 75^ *RAS* (*KRAS—*-Kirsten Rat Sarcoma viral oncogene homolog—chromosome 12p12.1 and *NRAS*—Neuroblastoma RAS viral oncogene homolog—chromosome 1p13.2) mutations are often associated with a MPN-like CMML phenotype.^76^ Although univariate analysis studies with *RAS* mutations have demonstrated inferior outcomes, these findings have not been substantiated in multivariable models.^7, 8^

The *CBL* gene (casitas B-cell lymphoma—chromosome 11q23.3) codes for an E3 ubiquitin ligase involved in degradation of activated receptor tyrosine kinases, thereby resulting in a negative modulation of tyrosine kinase signaling.^77^ RING finger domain mutations of *CBL* are frequently associated with UPD11q (uniparental disomy) and have been reported in 10–20% of patients with CMML.^7, 75, 77^ *CBL* mutations are associated with monosomy 7 and *TET2* mutations but, thus far, in CMML, have had no impact on OS or LFS.^7, 77^ *SH2B3* (SH2B adaptor protein 3, also called as *LNK*—chromosome 12q24.12) is a key negative regulator of cytokine signaling and has a critical role in hematopoiesis. SH2B3 directly binds to wild-type *JAK2* and *JAK2* V617F and decreases their autophosphorylation and downstream signaling through STAT5 (signal transducer and activator of transcription factor 5), MAPK (mitogen-activated protein kinase)/ERK (extracellular signal–regulated kinase) and the PI3K (phosphoinositide-3 kinase)/AKT pathways.^78^ *SH2B3* mutations are seen in ~5–7% of CMML patients and may co-occur with *CBL* mutations, suggesting a collaborative effect.^79^ These mutations, again, lack an independent prognostic effect on disease outcomes.

The *FLT3* gene (Fms-like tyrosine kinase 3—chromosome 13q12.2) codes for a type III receptor tyrosine kinase that regulates differentiation, proliferation and survival of hematopoietic progenitor cells. The *FLT3* ITD (internal tandem duplication) is found in ~30% of patients with cytogenetically normal AML and predicts poor outcomes.^80^ *FLT3* mutations (ITD and tyrosine kinase domain mutations) are seen in <5% of patients with MDS and CMML and, unlike in AML, do not impact OS or LFS.^7, 81^ Mutations involving *NPM1* (nucleophosphomin—chromosome 5q35.1) and *c-Kit* (chromosome 4q12) are very uncommon in CMML.^7^

## *SETBP1* mutations

*SETBP1* (SET-binding protein 1—chromosome 18q21.1) encodes the SET-binding protein 1, a binding partner for the multi-function SET protein. This protein is involved in apoptosis, transcription and nucleosome assembly.^82^ The proposed functional outcome of this interaction is based on *in vitro* studies that demonstrate a protection of SET protein from protease cleavage that results in inhibition of protein phosphatase 2A activity, leading to higher rates of cell proliferation. In CMML, *SETBP1* mutations have a frequency of 5–10%, with some^82, 83^ but not all studies demonstrating prognostic relevance.^6^

## Molecular and cytogenetic correlates in CMML

In a seminal, international collaborative study (Mayo Clinic and the French CMML consortium), cytogenetic and molecular correlates were assessed in 409 patients with WHO-defined CMML.^4^ The mutational frequency of commonly affected genes was: *SRSF2* (46%), *ASXL1* (37%), *U2AF1* (8%), *SF3B1* (7%), and *SETBP1* (4%), respectively. *ASXL1* and *SF3B1* mutations were associated with abnormal karyotypes (*P*=0.04 and *P*=0.03) and *SRSF2* with normal karyotypes (*P*=0.02).^4^ In comparison to other abnormal karyotypes, the incidence of *ASXL1* mutations was lower in -Y (*P*=0.04) and der(3q) (*P*=0.03). *U2AF1* mutations were associated with monosomal karyotypes (*P*=0.03) and *SF3B1* with der(3q) (*P*<0.0001).^4^ There were 9 (2%) patients with der(3q) abnormalities of which 6 had sole der(3q). Five of the 9 (55%) evaluable patients with der(3q) had *SF3B1* mutations and expressed BM RS. Iron stains were not available in four patients. This study resulted in the development of the aforementioned Mayo–French cytogenetic risk stratification system.

## Conclusions

CMML is a myeloid neoplasm with overlapping features of MDS and MPN, enriched with cytogenetic (~30%) and molecular abnormalities (>90%).^2, 22^ Common cytogenetic abnormalities include: trisomy 8, -Y, abnormalities of chromosome 7 (monosomy 7 and del7q), trisomy 21, del(20q) and complex karyotypes. The Mayo–French cytogenetic risk stratification system effectively risk stratifies CMML patients based on cytogenetic abnormalities.^4^ The advent of next-generation sequencing has identified multiple gene mutations in most CMML patients. These mutations tend to involve epigenetic regulator genes (*TET2*, *ASXL1*), splicing components (*SRSF2*), signal pathways (*RAS*, *CBL*) and transcription factors (*RUNX1*).^6, 7^ Among these, thus far, only nonsense and frameshift *ASXL1* mutations have been shown to negatively impact OS.^6, 7^ Expanding molecular insights into pathways altered by the above-mentioned genetic and epigenetic changes are slowly but surely translating into pharmacological interventions. A prime example is the availability of IDH inhibitors for *IDH1/2*-mutated myeloid neoplasms.^29^ Hopefully with time, molecular testing at diagnosis will not only help with disease prognostication but will also help offer better therapeutic approaches. The need of the hour is to develop a CMML prognostic model that incorporates cytogenetic and molecular abnormalities.

## Figures and Tables

**Table 1:** Cytogenetic and molecular correlates in patients with WHO-defined chronic myelomonocytic leukemia

*Cytogenetic abnormality*	*Frequency*	ASXL1 *mutational frequency*	SETBP1 *mutational frequency*	SRSF2 *mutational frequency*	U2AF1 *mutational frequency*	SF3B1 *mutational frequency*	*Spanish cytogenetic risk stratification*	*Mayo–French cytogenetic risk stratification*
*+8*	~23%	~24%	0%	~27%	0%	0%	High risk	Intermediate risk
*-Y*	~20%	~12% (SS)	~40%	~16%	0%	~9%	Low risk	Low risk
*-7/7q-*	~14%	~14%	~40%	~11%	40%	0%	High risk	Intermediate risk
*20q-*	~8%	~10%	0%	~11%	0%	0%	Intermediate risk	Intermediate risk
*der(3q)*	~8%	~2% (SS)	0%	~3%	20%	45% (SS)	Intermediate risk	Low risk
*+21*	~8%	~10%	0%	~5%	20%	0%	Intermediate risk	Intermediate risk
*Complex and monosomal karyotypes (MK)*	~10%	~4%	~20%	~8%	~40% (SS)	0%	Complex high risk. MK not included in stratification	High risk

Abbreviations: *ASXL1*, additional sex combs 1; CMML, chronic myelomonocytic leukemia; MK, monosomal karyotype; *SF3B1*, splicing factor 3B, subunit 1; *SRSF2*, serine/arginine-rich splicing factor 2; SS, statistically significant; *U2AF1*, U2 small nuclear RNA auxiliary factor 1; WHO, World Health Organization.
